# Anticancer Effects and Mechanisms of OSW-1 Isolated From *Ornithogalum saundersiae*: A Review

**DOI:** 10.3389/fonc.2021.747718

**Published:** 2021-09-23

**Authors:** Zhixin Zhan, Ziqiang Liu, Jiacheng Lai, Chaochao Zhang, Yong Chen, Haiyan Huang

**Affiliations:** Department of Neurosurgery, The First Hospital of Jilin University, Changchun, China

**Keywords:** OSW-1, synthesis, anticancer, effect, mechanism, future perspective

## Abstract

For centuries, cancer has been a lingering dark cloud floating on people’s heads. With rapid population growth and aging worldwide, cancer incidence and mortality are growing rapidly. Despite major advances in oncotherapy including surgery, radiation and chemical therapy, as well as immunotherapy and targeted therapy, cancer is expected be the leading cause of premature death in this century. Nowadays, natural compounds with potential anticancer effects have become an indispensable natural treasure for discovering clinically useful agents and made remarkable achievements in cancer chemotherapy. In this regards, OSW-1, which was isolated from the bulbs of Ornithogalum saundersiae in 1992, has exhibited powerful anticancer activities in various cancers. However, after almost three decades, OSW-1 is still far from becoming a real anticancer agent for its anticancer mechanisms remain unclear. Therefore, in this review we summarize the available evidence on the anticancer effects and mechanisms of OSW-1 *in vitro* and *in vivo*, and some insights for researchers who are interested in OSW-1 as a potential anticancer drug. We conclude that OSW-1 is a potential candidate for anticancer drugs and deserves further study.

## Introduction

Cancer, also known as malignant neoplasm, is a disease caused by abnormal cell growth and proliferation characterized as an uncontrolled cell division with the ability to metastasize. According to the GLOBOCAN 2020 by the International Agency for Research on Cancer, it was estimated that cancer has caused 19.29 million new cases with almost 10.0 million deaths (1). With rapid population growth and aging worldwide, cancer incidence and mortality are rapidly growing, and is expected to surpass cardiovascular disease as the leading cause of premature death in this century (2). Oncotherapy mainly includes surgery, radiation and chemical therapy, as well as immunotherapy and targeted therapy (3). Despite remarkable advances in medical technology, the cure rate and overall survival of cancer are still unsatisfactory in reality. Chemotherapy is the only option in majority of patients with advanced cancer, because surgical and radiation treatments are ineffective and traumatic. However, conventional chemotherapeutic drugs have great limitations. It may attack normal cells due to lack of selectivity to neoplastic cells, induce secondary malignancies during treatment of metastatic cancers, and develop drug resistance and high recurrence after treatment (4). Therefore, finding new anticancer drugs that are more effective, have multiple targets, and have low toxicity will become the breakthrough of chemotherapy. In this regard, natural compounds have a good potential.

In the 1920s, Berren et al. have begun to study the extracts of plants, marine organisms, and various microorganisms in search of natural anticancer compounds (5). Compared with conventional chemotherapeutic drugs, natural compounds have more diverse structures and excellent anti-tumor activity with low cytotoxicity. Traditional medicinal herbs and plants, which contain valuable bioactive compounds with potential therapeutic effects, have been an important source of several clinically useful anticancer agents; that was developed into standard approaches of tumor chemotherapy available today, such as vincristine for Leukemia, etoposide for small cell lung cancer and paclitaxel for ovarian and breast cancer (6). According to the statistical data released in 2016, from 1940s to the end of 2014, 49% of the 175 anticancer small molecular compounds approved by the US FDA were either natural products or their direct derivatives (7).

Ornithogalum saundersiae is a perennial herb bulbous plant belonging to the genus Ornithogalum of Liliaceae family, which is native to southern Africa and mainly planted in temperate regions of the Eastern Hemisphere. In the 1970s, O. saundersiae was introduced to China from Korea as an ornamental plant. In Chinese folk medicine, O. saundersiae is considered to have anti-inflammatory and antitumor properties, which has been used in therapy for hepatitis and some types of cancers (8). Scientific analyses have revealed that it contains more than 20 kinds of bioactive components, including saponins, polysaccharides, flavonoids, terpenoids, alkaloids, volatile oils and trace elements, and so on. In 1992, Kubo S et al. (9) isolated OSW-1, a steroidal saponins, from the bulbs of O. saundersiae, which has a high cytotoxicity to cancer cells. Its anticancer effect is about 10-100 times that of many chemotherapeutic drugs commonly used in clinic, such as doxorubicin, camptothecin and paclitaxel (10). The sensitivity of normal cells to OSW-1 is significantly lower than that of cancer cells; with the IC50 of OSW-1 is 40–150 folds higher than that observed in malignant cells, demonstrating its relatively high safety (11). However, the selective anticancer mechanism remains largely unclear, which limits further clinical applications. In this review, the anticancer effects of OSW-1 and its underlying mechanisms were summarized, in order to facilitate research to explore potential anticancer targets and prepare for its future clinical application.

### Synthesis and Structure Activity Relationship of OSW-1 and Its Derivatives

**Figure 1:**
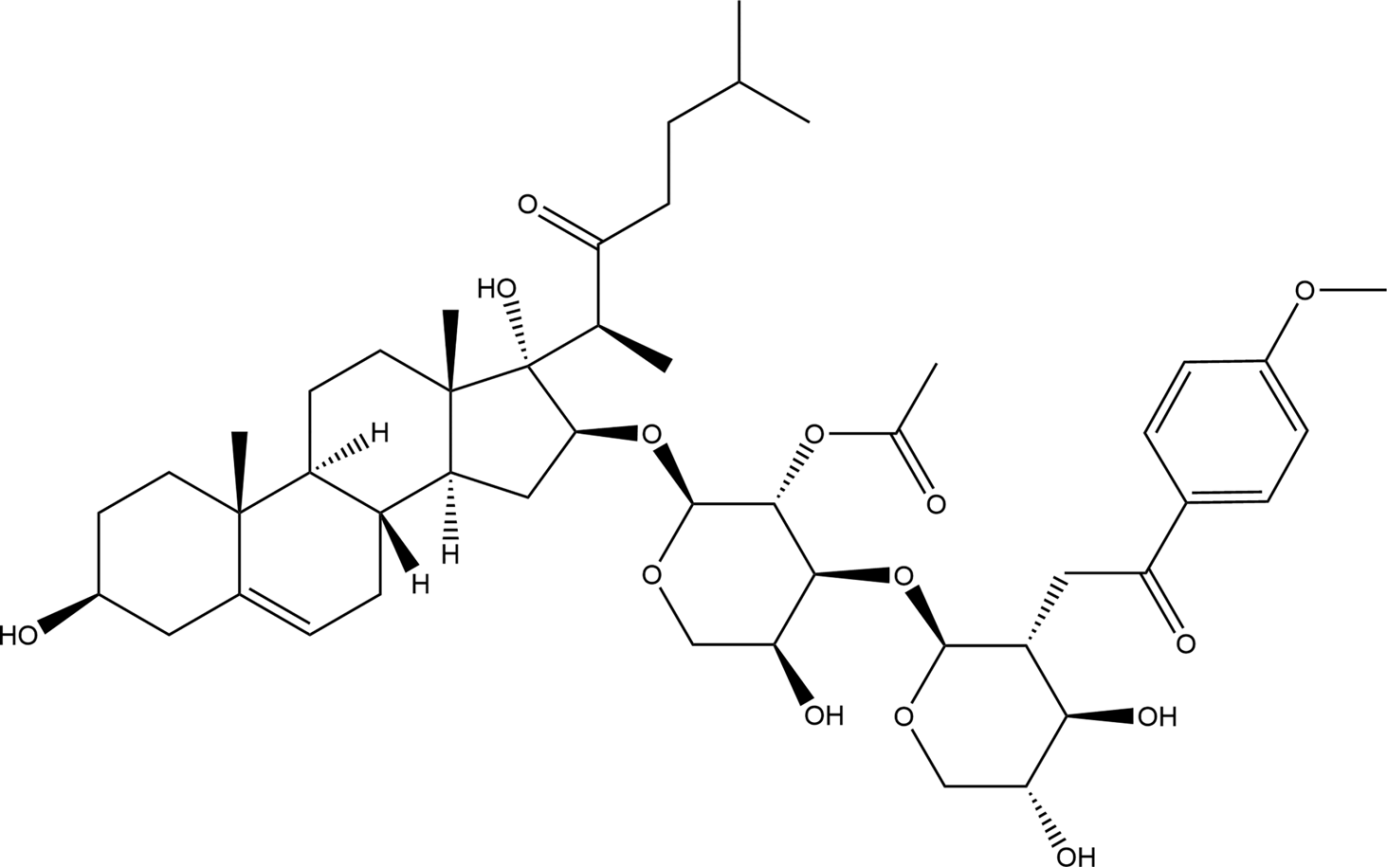
The chemical structure of OSW-1.

OSW-1 (C47H68O15), [IUPAC: [(2S,3R,4S,5R)-2-[(2S,3R,4S,5S)-3-acetyloxy-2-[[(3S,8R,9S,10R,13S,14S,16S,17S)-3,17-dihydroxy-10,13-dimethyl-17-[(2S)-6-methyl-3-oxoheptan-2-yl]-1,2,3,4,7,8,9,11,12,14,15,16-dodecahydrocyclopenta[a]phenanthren-16-yl]oxy]-5-hydroxyoxan-4-yl]oxy-4,5-dihydroxyoxan-3-yl] 4-methoxy benzoate], with molecular weight of 873.0 g/mol, is an acylated cholestane glycoside, which was first isolated by Kubo S et al. in 1992 (9). It was also proved by Mimaki Y et al. in 1997 (10), that it has an exceptionally cytostatic activity against various malignant tumor cells. **Figure 1** depicts the chemical structure of OSW-1. For the last three decades, the anticancer mechanisms of OSW-1 have remained unclear due to the extremely low acquisition rate by the traditional extraction methods and the relatively difficult chemical synthesis. Therefore, chemical synthesis of OSW-1 has been the subject of research. Recent studies have shown that the synthesis of OSW-1 and its derivatives is gradually improving (12–15).

The structure of OSW-1 can be divided into two parts: the cholestane aglycon and the disaccharide moiety. In 1998, Guo and Fuchs (16) first synthesized the protected aglycon of OSW-1 and confirmed that the anticancer activity of OSW-1 was due to the formation of oxygen cation intermediates between 22 oxygen and 16 β. Ma XQ et al. (17, 18) found that aglycone was an important structural component of OSW-1 to exert the activity by synthesizing a series of glycoside derivatives bearing the disaccharides moiety of OSW-1 and comparing their anticancer activities. In 1999, Deng S et al. (12) first synthesized OSW-1 by coupling of the aglycon with the sugar part from commercially available dehydroisoandrosterone, L-arabinose, and D-xylose, in total 27 steps with the longest linear sequence of 14 steps and with 6% yield. Yu and Jin (19, 20) used the same substrate but adopted a new strategy to synthesize 17 side chains by 1,4-Addition of an acyl anion equivalent to 17 (20)-en-16-one steroids, which greatly simplified the synthesis of OSW-1 into 10 steps with 28% yield. In 2002, Morzycki’s group (21) studied the direct glycosylation of the protected aglycone with the disaccharide trichloroimidate and found that the hydroxy-lactone 7 is a valuable intermediate in the synthesis of the highly potent cytostatic OSW-1. In 2005, Shi BF et al. (22) reported an aldol approach to the stereoselective construction of the 16R,17R-dihydroxycholest-22-one structure and provided a convenient route for the synthesis of the 23-oxa-analogue of OSW-1, which has an approximate anticancer activity to OSW-1. In 2008, Tsubuki’s group (23) embarked on the synthesis of OSW-1 in which thiophene ring 17 side chains was produced by employing 2,3-Wittig rearrangement reaction from the known 17E (20)-ethylidene-16α-hydroxy steroid. After a series of reactions, the final yield of OSW-1 was 15.6%. In the above synthesis process, the intermediates often need to be separated and purified before the next reaction was carried out, and the reaction conditions were not easily controlled, resulting in difficulties in the large-scale synthesis of OSW-1. To solve this problem, Xue et al. (24) developed a new practical synthetic method to synthesize OSW-1 on a gram scale, with an efficient procedure to prepare the sugar ligands and disaccharides, although the overall yield was just 6.4%. This synthesis is highlighted by the reliable transformations and the simplified workup procedures (25).

In addition, to better understand the SAR of OSW-1, some researchers have focused their work on the synthesis and activity comparison of its derivatives and have achieved some good results (13, 14, 26–29). For example, OSW-1 analogues with different modifications of hydroxyl (3β, 16α, 17β), disaccharide, 17-side chain and parent ABCD ring on OSW-1 can increase or decrease the overall activity. However, none of these studies have further explored the specific action targets of each functional group. In recent years, chemical probe-based approaches were proven powerful in the target identification studies of natural products (30). This may become a strong tool for studying the action targets of OSW-1 in the future.

## Anticancer Effects of OSW-1

### *In Vitro* Studies

In 1992, OSW-1 was found to be cytostatic in the U.S. National Cancer Institute 60-cell *in vitro* screen, with a mean IC50 of 0.78 nM and a mean IC100 of 58 nM (31). However, it was not until 1997 when Mimaki et al. (10) discovered that OSW-1 exhibited exceptionally potent cytostatic activities on various malignant tumor cells with little toxicity to normal cells that it began to attract the attention of researchers. Its anticancer effect is about 10-100 times that of doxorubicin, camptothecin and paclitaxel (10). Early studies have mainly focused on the synthesis of its derivatives and anticancer effects (14, 17, 18, 29, 32–34), because of the low field rate. Recently, studies on the anticancer mechanism of OSW-1 *in vitro* have gradually increased with the improvement of total synthetic process (12, 20, 22, 24, 34). OSW-1 has been shown to exhibit anticancer effect on various cancer cells, including ovarian, breast, cervical, colon, leukemia, hepatocellular carcinoma and other cancer cells. **Table 1** tabulates the *in vitro* studies on the efficacy and mechanisms of OSW-1 on different cancer cells (8, 10, 11, 35–43).

**Table 1:** The cytotoxic effects of OSW-1 against cancer cells in *in vitro* experiments.

References	Cell lines		Efficacy, IC50 (exposure time)	Mechanisms of action
(36)	Human ovarian cancer cell	SKOV-1(monolayer)	4.0 ± 2.7 nM(72h)	Anti-proliferation, the targeting of ORP4 is responsible for the anti-proliferative activity of the OSW-1 compound in the absence of exogenously supplied cholesterol
		OVCAR-1(monolayer)	2.2 ± 0.85 nM(72h)	
		OVSAHO(monolayer)	1.8 ± 0.61 nM(72h)	
		OVCAR-8(monolayer)	>1,000 nM(72h)	
		SKOV-3(spheroids)	10 nM(72h)	
		OVCAR-8(spheroids)	100 nM(72h)	
(37)	Human breast cancer cell	MCF-7	3.72 ± 0.78 nM(72h)	Inhibits tumor growth by inducing apoptosis, represses the migratory and invasive capabilities *via* EMT, inhibits tumor growth and metastasis by decreasing the expression of NFATc2
		T47D	5.92 ± 1.21 nM(72h)	
		ZR-75-1	10.34 ± 0.07 nM(72h)	
		BT474	6.54 ± 1.14 nM(72h)	
		SKBR3	6.67 ± 0.13 nM(72h)	
		MDA-MB-231	5.82 ± 2.35 nM(72h)	
		MDA-MB-453	8.66 ± 0.19 nM(72h)	
		HCC-1937	11.12 ± 4.42 nM(72h)	
	Human normal mammary epithelial cell	MCF/10A	52.3 ± 8.72 nM(72h)	N/A
(38)	Human cervical cancer cell	HeLa Cells	N/A	Induce mis localization of OSBP, which result in Golgi fragment and TFE3 activation, selectively trigger the apoptotic Golgi stress response *via* ↑CREB3-ARF4 proapoptotic pathway, ↓HSP47 antiapoptotic pathway
(39)	Human promyelocytic leukemia cells	HL-60 Cells	0.061 ± 0.0020nM(72h)	G2/M arrest, DNA fragmentation, caspase 3 activated, induce apoptosis *via* a mitochondria-independent signaling pathway
	Human lung adenocarcinoma cells	A549	0.65 ± 0.018nM(72h)	N/A
(8)	Human colon cancer cell	LoVo	31 ± 2.0 ng/ml(72h)	inhibition *via* induce intrinsic apoptosis, increased cellular calcium, changed mitochondrial membrane potential, disrupted mitochondrial morphology, release of cytochrome c and the activation of caspase-3
		SW480	61 ± 1.0 ng/ml(72h)	
	Human normal colonic mucosal epithelial cells		139 ± 9.0 ng/ml (72h)	N/A
(42)	Human leukemia cells	HL-60	the average IC50 value 0.019 nM(72h)	HL-60: disruption of cellular calcium homeostasis through inhibition of NCX1 and Inducing Apoptosis through a Mitochondrion-mediated Mechanism
		Raj		
		K-562		
		KBM5,		
		M1		
	Normal lymphocytes		the average IC50 value 1.64 nM(72h)	N/A
				
(41)	Human hepatocellular carcinoma cells	Hep3B	N/A	Induce apoptosis and necroptosis, inhibit invasiveness, angiogenesis, cell polarity and cell adhesion of cancer *via* ↓Wnt, ↓MAPK, ↓VEGF, ↑P53 signal pathways
(40)	Human hepatocellular carcinoma cells	Hep3B	N/A	Affect numerous miRNAs that act on specific signaling pathways for proliferation, differentiation, apoptosis, cell adhesion, migration and EMT
(43)	Human colon cancer cell	HCT-116	N/A	Anti-proliferation by targeting OSBP and ORP4L
	Chinese hamster ovary cells	CHO-7		
	human B cell lymphoma	M12 cells		
(44)	Chinese hamster ovary cells	CHO cells	N/A	Induce mitochondrial-mediated apoptosis pathway through caspase-8-dependent cleavage of Bcl-2
	Human acute T-lymphocyte leukemia cell	FADD and caspase-8-deficient Jurkat T cells		
(11)	Human leukemia cells	ML-1	0.19 nM(72h)	Damage the structure and function of mitochondrial and induce apoptosis through a calcium-dependent mechanism
		HL-60	0.044nM(72h)	
	Human lymphoma cell	Raji	0.58nM(72h)	
	Human ovarian cancer cell	SKOV3	0.021nM(72h)	
	Human glioblastoma cell	U87-MG	0.047nM(72h)	
	Human pancreatic cancer cells	AsPC-1	0.0391nM(72h)	
	Nonmalignant cells	normal lymphocytes	1.73nM(72h)	
		ovary fibroblasts	0.83nM(72h)	
		normal brain astrocytes	7.13nM(72h)	
(10)	Human normal pulmonary cell	CCD-19Lu	1.5μg/ml(N/A)	N/A
	Mouse leukemia	P388	0.00013μg/ml(N/A)	
	Adriamycin-rcsistant P388	P388/ADM	0.00077μg/ml(N/A)	
	Camptothecin-resistant P388	P388/CPT	0.00010μg/ml(N/A)	
	Mouse microcarcinoma	FM3A	0.00016μg/ml(N/A)	
	Human pulmonary adenocarcinoma	A549	0.00068μg/ml(N/A)	
	Human pulmonary large cell Carcinoma	Lu-65	0.00020μg/ml(N/A)	
	Human pulmonary large cell Carcinoma	Lu-99	0.00020μg/ml(N/A)	
	Human pulmonary squamous cell	RERF-LC-AI	0.00026μg/ml(N/A)	
	Carcinoma	CCRE-CEM	0.00016μg/ml(N/A)	
	Human leukemia	HL-60	0.00025μg/ml(N/A)	

NA, Not available; ↑, upregulation ; ↓, downregulation.

As shown in **Table 1**, OSW-1 has a high selective cytotoxicity to cancer cells compared with normal cells. It suggested that OSW-1 is expected to be developed into a new anticancer drug with the potential to specifically kill cancer cells. Although different cancer cells have different sensitivity to the inhibition effect of OSW-1, all of their IC50 values are in the nanomolar concentration range. Furthermore, OSW-1 appears to exert anticancer effects in cancer cells through different mechanisms, since different type of cancers have their unique key action targets. OSW-1 happens to be a natural compound with multiple anticancer targets due to its complex structure, which can regulate various signaling pathways (40, 41) and inhibit the development and progression of cancer cells by arresting cell cycle (38), damaging the structure and function of mitochondria, disrupting the cellular calcium homeostasis, inducing apoptosis (8, 11, 36, 38, 39) and Golgi stress response (37), inhibiting proliferation (35, 42) and metastasis, and repressing the migratory and invasive capabilities *via* EMT (36). Interestingly, necrosis was also detected when cells were treated with a high dose (180 ng/ml), which means OSW-1 may mediate other cell death pathways (8). Overall, OSW-1 exhibits potent anticancer potential against different cancer cells *in vitro*.

### *In Vivo* Studies

Based on some *in vivo* studies, OSW-1 has been proved to be effective in inhibiting tumor growth, such as breast cancer, colon cancer, and leukemia (**Table 2**) (8, 10, 36). In 1997, Mimaki et al. (10) found that OSW-1 was remarkably effective versus mouse P388, with an increased life span of 59% by only one time administration of 0.01 mg kg -1. However, they did not further explore the specific mechanisms behind this effect. For a long time afterwards, the OSW-1 seemed to disappear from the researchers’ view. Until recently, Zhang et al. (8) and Ding et al. (36) began to investigate the anticancer effect of OSW-1 *in vivo* and explored the possible mechanism, respectively. To ascertain whether or not OSW-1 was as effective *in vivo*, Zhang et al. (8) adopted heterotopic xenograft tumor model in nude mouse subcutaneously inoculated by LoVo cells, in which OSW-1 was injected intraperitoneally (0.01 mg/kg diluted in PBS in 500 µ, daily) in treated group when tumors became palpable. Compared with the control group, the treated group observed a decrease in tumor size and weight without significant side effects, with fewer Ki-67-positive cells and more apoptotic cells. Interestingly, they also observed a destruction of blood vessels and a reduction in angiogenesis pathologically in the treated group. It suggested that OSW-1 may be involved in reduction in angiogenesis and tumor metastasis.

**Table 2:** The anti-cancer effects of OSW-1 *in vivo* tumor bearing animal models.

References	Animal models		Dose, duration and route of administration	Observations and results	Mechanisms of action
(36)	Human breast cancer cell	MDA-MB-231 xenograft model	0.01 mg/kg diluted in 100 μL PBS, daily, 20 days, ip	Reduction of tumor size and weight, Ki67↓, PCNA↓	Inhibits tumor growth
		MDA-MB-231 orthotopic model	0.01 mg/kg diluted in 100 μL PBS, until the tumors in control group reach 1.0 cm, continue injecting OSW-1 for 1 week, ip	Fewer metastatic nodules in lungs and longer survival, E-cadherin↑ and ↓ Vimentin	Inhibits metastasis mediated by EMT
		knockdown NFATc2 model	0.01 mg/kg diluted in 100 μL PBS, daily, 20 days, ip	Knocking down of NFATc2 using shRNA significantly rescues TNBC cells from OSW-1-mediated effects on cell death, tumor growth, invasion and migration	NFATc2 is involved in OSW-1 inhibition of TNBC progression.
(8)	Human colon cancer cell	LoVo xenograft model	0.01 mg/kg diluted in PBS in 500 µl, daily, 21 days, ip	Reduction of tumor size and weight, no apparent side effects, ki-67↓, no necrotic foci, induce apoptosis	Suppressing colon tumor proliferation without significant side effects through the apoptosis pathway
(10)	Mouse P388 leukemia cell	P388 cell intraperitoneal implantation model	0.01 mg/kg, one time, N/A	Increased life span of 59%	N/A

NA, Not available; ↑, upregulation; ↓, downregulation; i.p., intraperitoneal administration.

In 2020, Ding et al. (36) designed a series of experiments to verify the effect of OSW-1 on the tumor growth and metastasis of breast cancer, including three innovative animal models of tumor *in vivo*. They have found the following (1): for xenograft model, OSW-1 can inhibit tumor growth with reduction of tumor size and weight (2); for orthotopic model, fewer metastatic nodules in the lungs and longer survival were observed in treated group, with downregulation of Vimentin and upregulation of E-cadherin, which means OSW-1 can inhibit metastasis mediated by EMT; and (3) for knockdown NFATc2 model, identified NFATc2 may be a pivotal factor for OSW-1-mediated effects on cell death, tumor growth, invasion, and migration.

Overall, OSW-1 has good anticancer properties *in vivo*, and it is worthy of further research in the field of cancer chemotherapy. However, this requires more *in vivo* experiments to prove that OSW-1 can also exert similar anticancer effects in other cancers besides breast and colon cancer. The subsections below will further discuss the anticancer mechanism of OSW-1.

## Anticancer Mechanisms of OSW-1

### OSW-1 Inhibits the Proliferation of Cancer Cells

The most fundamental trait of cancer cells is the ability to proliferate and grow without limit. Cancer cells become masters of their own destinies by inducing and sustaining positively acting growth-stimulatory signals, which allow them to enter the proliferation and growth cycles, incessantly (44). Although there is still insufficient knowledge about the precise mechanism controlling the proliferative signals of cancer cells, dysregulation of the cell cycle is considered to be the main contributor to uncontrolled cell proliferation (45). Therefore, cell cycle inhibitors are becoming attractive targets in cancer treatment. It was found that the cell cycle was arrested at the G2/M phase in HL-60 cells following treatment with OSW-1 at concentration of either 0.3 nM or 0.01 µM (38). In addition, Jin et al. (40) examined the potential changes in the gene expression of a hepatocellular carcinoma cell line incubated with OSW-1 *in vitro*, and performed the enrichment analysis of the differentially expressed gene on signaling pathways. The results showed that the cell cycle is ranked first in enrichment score, which mean OSW-1 greatly affects the expression of cell cycle-related genes.

Oxysterol-binding protein (OSBP) and its related proteins (ORPs) constitute a large, evolutionarily conserved family of lipid-binding proteins that mediate signal transduction and lipid transport. It is reported that the OSBP/ORPs family is implicated in cell proliferation and cancer development (46, 47). Therefore, OSBP/ORPs may be potential therapeutic targets in cancer. In 2011, Burgett et al. (42) first identified that OSBPs and ORP4 are high-affinity receptors of OSW-1 and can mediate the anti-proliferative activity of OSW-1 in cancer cells. The activity was also recognized in ovarian cells by Bensen et al. (35). Notably, the cytotoxicity of OSW-1 was consistent with the reduction of the ORP4 expression, but not with the reduction of OSBP expression, suggesting ORP4 is the main anti-proliferative target of OSW-1 (35). Recently, some studies show that OSBP does not have any known role in cellular proliferation (48, 49); while ORP4 participate in the control of human malignant tumor cell proliferation and survival (50, 51). Interestingly, in the absence of extracellular lipids, OSW-1 has enhanced antiproliferative activity and OSBP, but not ORP4, is likely responsible for the striking shift in sensitivity to OSW-1 (35). OSBP is reported to be required for lipid transport by burning off the phosphoinositide phosphatidylinositol 4-phosphate [PI (4)P] between the endoplasmic reticulum (ER) and the Golgi (52), and dysregulation of PI4P metabolism and protein interactions are often associated with tumor progression and a poor prognosis (53). Thus, it is confused whether OSBP or ORP4 take more charge of the antiproliferative activity of OSW-1, or maybe both, which require further investigation.

### OSW-1 Induces Apoptosis of Cancer Cells

Apoptosis, a form of programmed cell death that results in the orderly and efficient removal of damaged cells, occurs during development as a homeostatic mechanism to maintain cell populations in tissues, and as a defense mechanism when cells are damaged by harmful stimulations (54, 55). Inappropriate apoptosis is involved in many human diseases, including neurodegenerative diseases, ischemic damage, and autoimmune disorders (56). In cancer, minimal apoptosis results in malignant cells proliferation without limitations (57). The mechanisms of apoptosis can be divided into two main pathways: the intrinsic pathway, which is mediated by the mitochondria; and extrinsic pathway, which is mediated by death receptors, including FasL/FasR and TNF-α/TNFR1 (58). These two apoptotic pathways both involve activation of series of caspases and converge on the same execution pathway, which is initiated by the cleavage of caspase-3, leading to morphological and biochemical cellular alterations that are characteristics of apoptosis (57, 59). The upstream caspase for intrinsic pathway is caspase 9, while that of extrinsic pathway is caspase 8 (57). With the deepening understanding on the regulatory mechanism of apoptosis, drugs that target the deregulated apoptotic pathways to promote apoptosis has become an important strategy for chemotherapy (60).

OSW-1 has been known to effectively induce apoptosis in different cancers. In breast cancer, Ding et al. (36) confirmed that OSW-1 was capable of inducing apoptosis by using Annexin V/PI-labeled flow cytometry and TUNEL assay and discovered that the expression levels of cleaved caspase-3 and cleaved PARP increased in a dose-dependent manner. Furthermore, apoptosis was also observed in other cancer cells including colon cancer (8), leukemia (11, 38, 39, 43), pancreatic cancer (11), and cervical cancer cells (37), though the specific mechanisms were not exactly the same (**Table 1**).

OSW-1 can initiate apoptosis through the intrinsic pathway. In colon cancer, Zhang et al. (8) discovered that OSW-1 damaged the structure and function of mitochondria leading to the release of cytochrome c that caused caspase-3 activation, which was regarded as the classical intrinsic apoptotic pathway. In the two studies by Zhou et al. (43) and Garcia et al. (39), they all suggested that OSW- 1 reduced mitochondrial membrane potential and then induced mitochondria-mediated apoptosis. Notably, the overload of cytoplasmic calcium was found in those studies and regarded to play a key role in cell death. Zhou et al. (11) thought that the damaged mitochondria leading to the calcium imbalance. Garcia et al. (39) hold the view that the inhibition of NCX1 (sodium-calcium exchanger 1) by OSW-1 was the reason for cytoplasmic calcium to lose homeostasis leading to calcium overload.

Besides the intrinsic pathway, OSW-1 is capable of inducing the extrinsic pathway. Lguchi et al. (38) found that OSW-1 induced apoptosis in HL-60 cells *via* the mitochondria-independent pathway, for no disruption of the mitochondria membrane potential and release of cytochrome C was observed. Zhu et al. (43) showed that OSW-1 induces apoptosis *via* caspase-8-dependent cleavage of Bcl-2 in Chinese hamster ovary cells. Furthermore, Jurkat T cells deficient in caspase-8 or FADD were resistant to apoptosis induced by OSW-1, which suggested that the extrinsic pathway is involved in the OSW-1-induced apoptosis (43). Bcl-2, an anti-apoptotic member of Bcl-2 family, once cleaved, will amplify the apoptotic signal through the mitochondria by altering its membrane permeability to facilitate the release of apoptogenic proteins such as cytochrome C (61).

OSW-1 can also induce apoptosis by Golgi stress-induced mechanism. In 2019, Kimura et al. discovered that OSW-1, as a novel class of selective Golgi stress inducer, can regulate Golgi stress response pathways, in which HSP47 was downregulated and CREB3-ARF4 was upregulated (37). It’s reported that the suppression of HSP47 under a Golgi stress condition leads to caspase 2-mediated apoptosis (62). In addition, CREB3-ARF4 mediates pro-apoptotic pathways in response to Golgi stress was also demonstrated by Reiling et al. (63).

**Figure 2:**
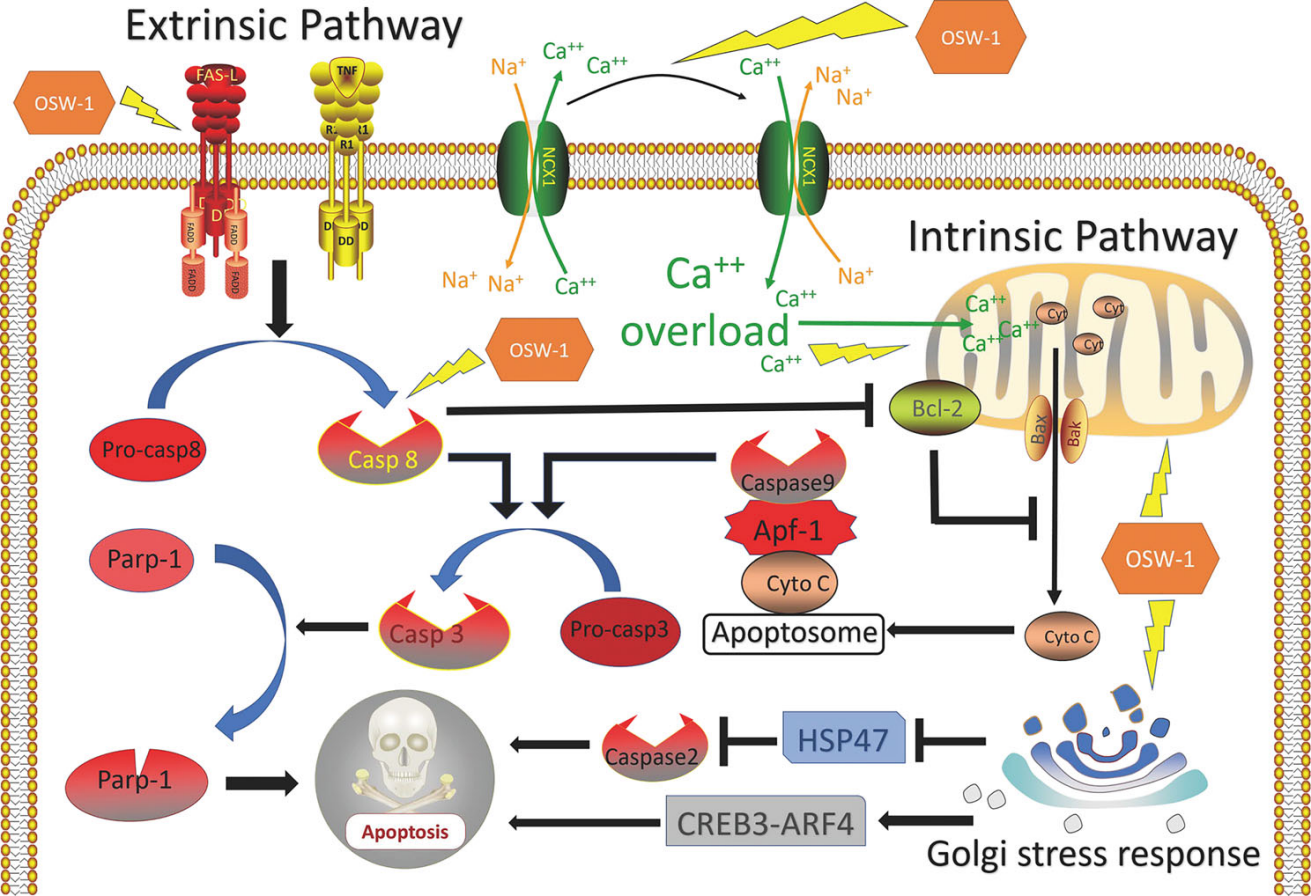
Overview of mechanisms of apoptosis induced by OSW-1 in cancer cells.

In summary, OSW-1 has the ability to promote apoptosis in cancer cells by activating various apoptotic pathways (**Figure 2**).

### OSW-1 Induces Golgi Stress Response of Cancer Cells

The classical view of Golgi apparatus is a small membranous organelle involved in protein transport and glycosylation (64). Recent descriptions of Golgi network demonstrate the essential role of Golgi in cellular activities, including mitosis, DNA repair, stress responses, cell death, and cancer development (65, 66). Changes on Golgi trafficking, signaling, and morphology in some malignant cancers were so obvious that the term ‘onco-Golgi’ has been proposed to describe those particular changes (67). Thus, the Golgi should have a fundamental impact on cancer cell survival and emerge as a new cancer therapeutic target. The Golgi stress response is an autoregulated mechanism for maintaining the homeostasis of Golgi apparatus similar to ER stress response (68) by regulating specific functions and size of various zones of the Golgi apparatus, especially zones related to apoptotic signaling pathway in accordance with cellular demands (69). In short, if the capacity of Golgi function becomes insufficient after various cellular stresses, Golgi will activate the response signaling pathways (70). Normally, Golgi stress response should serve to help alleviate the stress, and only result in cell death if the stress is harmful and irreparable.

Recently, several pathways of the mammalian Golgi stress response have been identified, especially the TFE3, HSP47, and CREB3-ARF4 (71). In 2019, Kimura et al. (37) discovered that OSW-1 preferentially localizes to the Golgi apparatus and activates the major Golgi stress response pathways by inducing mis-localization of OSBP from cytoplasm to the trans-Golgi network, which lead to the activation of TFE3 and the fragment of Golgi, and then selectively triggering the apoptotic Golgi stress response *via* upregulating CREB3-ARF4 proapoptotic pathway and downregulating HSP47 antiapoptotic pathway. Although OSW-1 induction of the selective Golgi stress response in cancer cells remains to be explored, their study provided the first evidence that link OSW-1-OSBP interactions with cell death induction (37). Overall, OSW-1 can effectively kill cancer cells by inducing Golgi stress response-mediated apoptosis, which provide guidance and reference for clinical development of novel anticancer drugs targeting Golgi apparatus.

### OSW-1 Suppresses Migration, Invasion, and Angiogenesis of Cancer Cells

Metastasis is the process by which cancer cells leaves the primary site, travels to distant regions *via* the circulatory system, and establishes a secondary tumor (72). It causes most cancer deaths and involves migration, invasion and angiogenesis, which are broadly regulated by epithelial-mesenchymal transition (EMT) (44). EMT is a biological process in which epithelial cells are converted into cells with mesenchymal phenotype. In cancer, it is also known as epithelial cell plasticity and is associated with tumor initiation, invasion, metastasis, and resistance to therapy (73). It usually begins with the disappearance of epithelial cell polarity and the weakening of intercellular adhesion (74). The loss of epithelial markers, such as cytokeratins and E-cadherin, and the acquisition of mesenchymal markers, such as N-cadherin and vimentin, indicate that the cancer cells gain the ability to migrate and invade. Therefore, blocking EMT of cancer cells will greatly reduce the metastasis rate, thus improving the prognosis of cancer patients.

Triple negative breast cancer (TNBC) is a particularly aggressive subtype of breast cancer and accounts for 15% to 20% of cases (75). It is characterized by a lack of expression of estrogen, progesterone, and human epidermal growth factor 2 receptors and has clinical features that include high invasiveness, high metastatic potential, proneness to relapse, and poor prognosis (76). In 2020, Ding et al. found that the OSW-1 decreased the expressions of NFATc2, and inhibited migration and invasion of TNBC cells *via* blocking the EMT signaling pathways *in vitro* and *in vivo* experiment (36). It was reported that nuclear factor of activated T cells (NFAT) is associated with TGFβ-induced EMT, which could influence proliferation, invasion, migration and angiogenesis of cancer cells (77).

In 2017, Zhang et al. (8) found destruction of blood vessels, reduction of angiogenesis and no metastatic focus in xenograft model with OSW-1 treatment, suggesting that OSW-1 may be involved in angiogenesis and tumor metastasis. The authors did not explore the mechanism behind this phenomenon. It was hypothesized that OSW-1 induce Golgi stress response and lead to the fragment of Golgi apparatus (37), which disrupt the homeostasis of PI4P and PI4P-binding proteins, including GOLPH3 or PITPNC1. However, these proteins are essential to the development of aggressive metastatic and invasive tumor for their ability to induce malignant secretory phenotype conversion leading to the release of proteins that can reshape the extracellular matrix, promote pathological angiogenesis, and enhance cell migration (53). The dysregulation of miRNA and signaling pathways caused by OSW-1 may also contribute to cancer metastasis and angiogenesis as discussed.

### Effect of OSW-1 on miRNAs Expression

MicroRNAs (miRNAs) are a major class of small noncoding RNA that consists of approximately 20 nucleotides, which negatively regulate gene expression at the mRNA level, usually silencing genes by binding to the 3′ or 5′‐untranslated region (UTR) of their target mRNAs, controlling genes involved in cellular processes, such as inflammation, proliferation, cell-cycle regulation, stress response, differentiation, programed cell death, and migration (78, 79). The cancer‐related miRNAs can be divided into two groups: tumor suppressor miRNAs (inhibiting tumor progression by targeting mRNAs that code oncoproteins and repressing the translation of oncogenic mRNAs) and oncogenic miRNAs (promoting tumor progression by promoting metastasis and silencing the tumor suppressor genes) (80). Given that miRNAs play roles in almost all aspects of cancer biology, and dysregulation of miRNAs is common in many cancers, it has been suggested that miRNAs could serve as potential tumor markers for the diagnosis of cancer, and developing new molecules targeting miRNAs expression in cancer represents an attractive strategy for oncotherapy (81, 82).

In 2013, Jin et al. (41) identified differential miRNA expression of Hep3B with OSW-1 treated *in vitro*, and the results showed that OSW-1 regulated many miRNAs, in which miRNA-142, miRNA-299, miRNA-187, miRNA-210, miRNA-125b and miRNA-200c were upregulated, and miRNA-126 was downregulated. Then, authors connected and identified functions of differential miRNAs with unrecognized functions of OSW-1, and drew a conclusion that OSW-1 inhibits cancer by affecting numerous cancer‐related miRNAs that acts on specific signaling pathways for proliferation, angiogenesis, apoptosis, cell adhesion, migration, and EMT (41). For instance, miRNA-126, an endothelial cell restricted miRNA, is associated with tumor angiogenesis for its ability to enhance pro-angiogenic actions of VEGF and FGF (83). After OSW-1 treatment, the expression of miRNA-126 decreased significantly (barely detected), which reduced the ability of tumor angiogenesis and led to the inhibition of tumor growth (41). Overall, the effect of OSW-1 on regulating miRNAs deserved further exploring.

### Effect of OSW-1 on Various Signaling Pathways

Signaling pathway, also called signal transduction, is a series of enzymatic reaction pathways that can transmit extracellular molecular signals into cells through cytomembrane to exert effects. With the deeper understanding of the molecular basis of neoplastic cell behavior, cancer is considered as a disease with altered signal pathways (84). Currently, signal pathway inhibition by blocking the enzymes and growth factor receptors that are essential for cell proliferation are being explored. Some have achieved remarkable success and are now commonly used as anticancer drugs, such as gefitinib for non-small cell lung cancer (85), imatinib for chronic myeloid leukemia (86) and trastuzumab for breast cancer (87). Therefore, agents that directly block pathogenic signal pathways by targeting key components to inhibit growth of cancers are a promising therapeutic strategy.

However, all targeting drugs have a common limitation which is the inevitable emergence of drug resistance (88–90). The reason is very simple, cancer cells are not easily killed by blocking one target in a single way, since the signal pathways in them are very complex and cross-talk and usually, when one of the pathways is blocked, another compensatory bypass will be activated (91). In this regard, natural products with potential multiple targets may be a solution (92). In a study of Jin et al. (40), the potential gene expression changes of Hep3B incubated with OSW-1 *in vitro* were examined and results showed that OSW-1 affected the expression of core genes in a number of signaling pathways, including the downregulation of Wnt, MAPK, and VEGF, and upregulation of P53. Wnt signaling pathway is important for its crucial function in development and growth, and has also been tightly associated with cancer for its aberrant activation involved in maintenance of cancer stem cells, metastasis and immune control (93); MAPK signaling pathway represents ubiquitous signal transduction pathways that regulates cell growth, differentiation, proliferation, apoptosis and migration functions, and play a role in tumorigenesis and associated with anticancer drug resistance (94); EGFR is a group of transmembrane proteins with cytoplasmic kinase activity and is frequently mutated and/or overexpressed in human cancers (95), which results in increased cell proliferation, abnormal metabolism, and cell survival through the activation of the downstream signaling pathways, such as MAPK, AKT, and STAT3 (96, 97); P53, a tumor-suppressor gene, is activated by a host of stress stimuli and, in turn, induce cell cycle arrest or apoptosis programs to inhibit cancer (98). Although OSW-1 inhibit cancer cells through regulating above signaling pathways require more rigorous verification, it also provides a new perspective to demonstrate the anticancer mechanisms of OSW-1.

## Future Perspective

In 1992, OSW-1 was first isolated from O. saundersiae and emerged as a candidate of anticancer drugs for its more powerful anticancer effect than doxorubicin, camptothecin and paclitaxel (9). Currently, many *in vitro* and *in vivo* studies have identified the anticancer effects of OSW-1 in various cancers and explored the potential targets. However, after almost thirty years, the anticancer mechanisms of OSW-1 are still undefined. Although Burgett et al. revealed that OSW-1 exhibits cytotoxicity by targeting OSBP and ORP4L (42), the link between these targeting and apoptosis induction has remained unclear. Furthermore, the OSBP-OSW-1 interaction seem to have more applications in antiviral than anticancer for OSBP is not essential to cell viability (99) but indispensable to virus replication (48, 100). In addition, the OSBPs targeting is hard to explain especially the high selective cytotoxicity of OSW-1. In 2013, Garcia et al. (39) demonstrated that OSW-1 inhibited NCX1 in a fashion similar to the NCX inhibitor KB-R7943, and then disrupted calcium homeostasis of cytoplasm leading to mitochondria-mediated apoptosis. Interestingly, this inhibitory effect of OSW-1 is cancer cell-specific with minimal effect on normal lymphocytes. An earlier study by Harley et al. (101) also found that the NCX inhibitors selectively kill malignant glioma cells but not primary astrocytes. Thus, future studies should concentrate on uncovering the precise key target proteins of OSW-1 to explain the high selective cytotoxicity.

Nowadays, chemical probe-based approaches have emerged as powerful methods for mechanistic studies of natural products by identifying the cellular site of action, the target proteins, and the target cellular pathways. However, studies on the development of chemical probes of OSW-1 for investigation of its biological role are still lacking (30). Recently, network pharmacology approaches for predicting unexplored targets and therapeutic potential are being applied increasingly to find new therapeutic opportunities of natural products (102). These two methods may be beneficial to reveal intracellular localization properties of OSW-1 and discover target proteins leading to the phenotypes of interest. In addition, the surge of “-omics” technologies, including genomics, transcriptomics, epigenomics, proteomics, metabolomics, has enabled us to recognize biological and molecular changes underlying the development and progression of human disease, and multi-omics analyses, which take advantage of these technologies (103, 104), may facilitate the clinical application of OSW-1 in precise treatment of cancer.

Given the low yield from extraction and limited amount present in raw plant materials, it is crucial to find a new way to synthesize OSW-1 in large quantities. Several groups have achieved total synthesis of OSW-1 and a number of its analogues have been synthesized to decipher the SAR (25), but the progress is slow. Comparing total synthesis, exploring derivatives with simplified molecular structure, with a more potent anticancer effects, would be a good strategy taking the complex structure of OSW-1 in consideration. In addition, with the advent of genetic engineering, the mass production of OSW-1 from plants using gene editing is also an option (105).

Notably, the pharmacokinetics of OSW-1, which involves the study of drug movement within the body, including the time course of absorption, distribution, metabolism, and excretion, is a blank of current researches. Moreover, although normal cells are less sensitive to the cytotoxicity of OSW-1, their IC 50 value is in nanomolar range, indicating that OSW-1 is still toxic to them (11). Thus, it is essential to perform vigorous animal toxicology experiments before considering evaluation of clinical application. In fact, toxicity and drug-like properties have become one of the main obstacles for many saponin drugs, including osw-1, to further move to clinical application, despite their extensive research and remarkable anticancer effects (106). However, only a few studies of OSW-1 involve in toxicology *in vivo* experiments (8, 10, 36). It is recommended that more *in vivo* experiments be performed to refine the pharmacokinetics and pharmacology of OSW-1, so that actual metabolites and concentrations are taken into account during *in vitro* experiments to simulate more realistic *in vivo* conditions. In addition, exploring more reasonable combination therapy regimens and more effective drug delivery systems to ensure increased efficacy and decreased toxicity of OSW-1 will also be one of the focuses of future research.

## Conclusion

**Figure 3:**
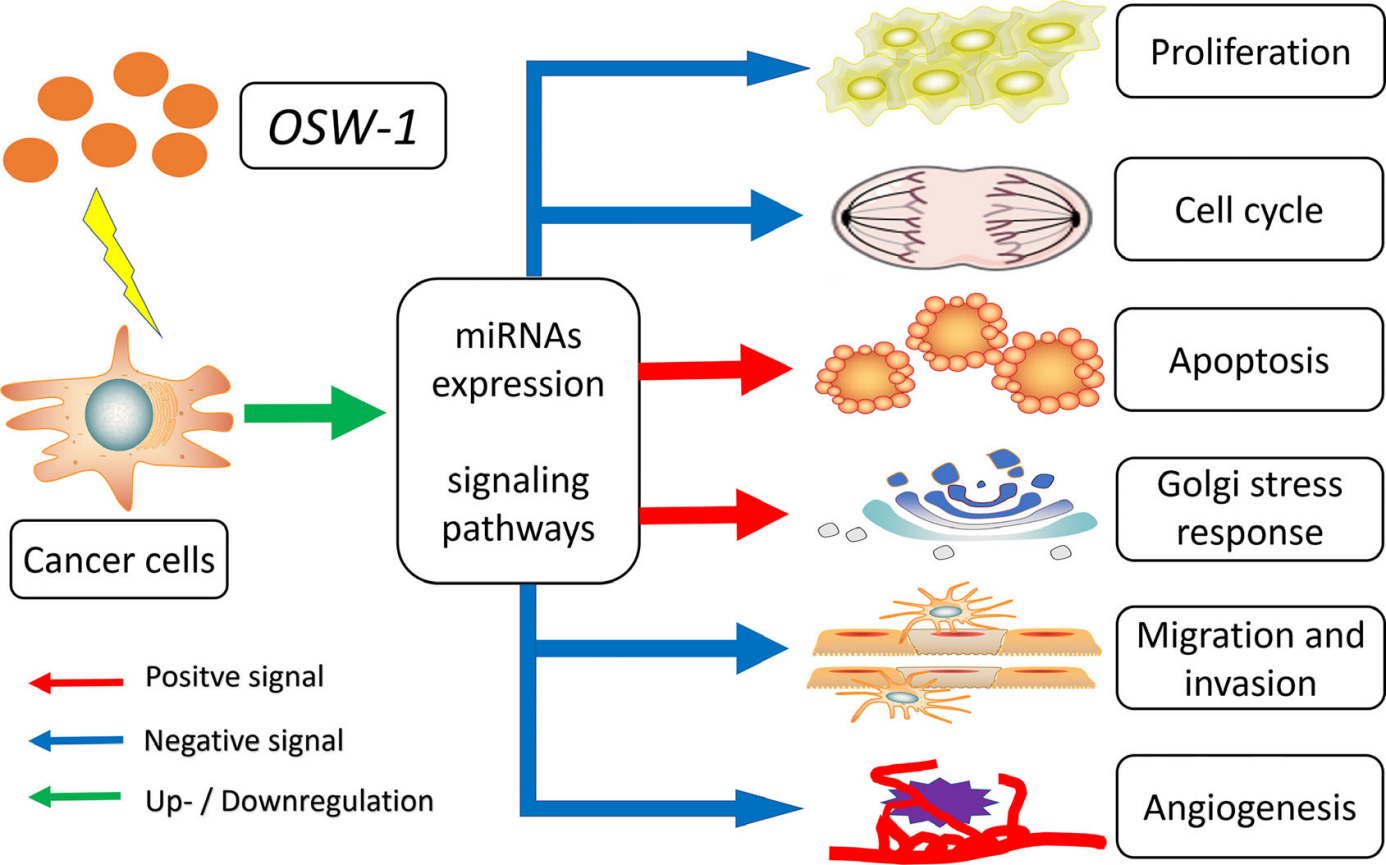
Overview of the anticancer mechanisms of OSW-1 in cancer cells.

It has been almost 30 years since OSW-1, a natural compound with potent anticancer activity, was first discovered in 1992. Currently, an increasing number of preclinical studies have confirmed the role of OSW-1 in anticancer therapies. Summarized in this review are the available evidence on the anticancer effects and mechanisms of OSW-1 *in vitro* and *in vivo*. As mentioned, OSW-1 has been shown to repress cancer progression through inhibiting cell proliferation, arresting cell cycle, inducing apoptosis and Golgi stress response, as well as suppressing migration, invasion and angiogenesis by regulating miRNAs expression and various signaling pathways (**Figure 3**). However, OSW-1 is still far from becoming a real anticancer agent for some issues, including anticancer mechanisms that have not been fully explained, especially the high selective cytotoxicity to cancer cells, the low yield rate from extraction and synthesis, and the need for more vivo experiments to refine pharmacokinetics.

In general, OSW-1 is a potential candidate for anticancer drugs and deserves further study. Since there are still some problems to be solved before it can be used in clinical treatment, this will require the joint efforts of different professional scientists, worldwide.

## Author Contributions

The manuscript was written by ZZ. The review of literature and data collection were performed by ZL, JL, and CZ. The manuscript was critically reviewed and edited by YC. The project was conceptualized by HH. All authors contributed to the article and approved the submitted version.

## Funding

This work was supported by Scientific Research Foundation of Jilin province (20180101158JC, 20200201613JC, 20200201388JC, 20190701042GH), Research and Planning Project of the 13th Five-Year Science and Technology Project of Jilin Provincial Department of Education (JJKH20180191KJ) and Interdisciplinary Innovation Project of First Hospital of Jilin University (JDYYJC001).

## Conflict of Interest

The authors declare that the research was conducted in the absence of any commercial or financial relationships that could be construed as a potential conflict of interest.

## Publisher’s Note

All claims expressed in this article are solely those of the authors and do not necessarily represent those of their affiliated organizations, or those of the publisher, the editors and the reviewers. Any product that may be evaluated in this article, or claim that may be made by its manufacturer, is not guaranteed or endorsed by the publisher.
